# Reducing hospital admissions from nursing homes: a systematic review

**DOI:** 10.1186/1472-6963-14-36

**Published:** 2014-01-24

**Authors:** Birgitte Graverholt, Louise Forsetlund, Gro Jamtvedt

**Affiliations:** 1 Centre for Evidence-Based Practice, Bergen University College, Bergen PB 7030, 5020, Norway; 2 Department of Global Public Health and Primary Care, University of Bergen, Bergen, Norway; 3 The Norwegian Knowledge Centre for the Health Services, Oslo, Norway

**Keywords:** Nursing home, Homes for the aged, Hospitalization, Hospitalisation, Acute care, Hospital admission

## Abstract

**Background:**

The geriatric nursing home population is vulnerable to acute and deteriorating illness due to advanced age, multiple chronic illnesses and high levels of dependency. Although the detriments of hospitalising the frail and old are widely recognised, hospital admissions from nursing homes remain common. Little is known about what alternatives exist to prevent and reduce hospital admissions from this setting. The objective of this study, therefore, is to summarise the effects of interventions to reduce acute hospitalisations from nursing homes.

**Methods:**

A systematic literature search was performed in Cochrane Library, PubMed, MEDLINE, EMBASE and ISI Web of Science in April 2013. Studies were eligible if they had a geriatric nursing home study population and were evaluating any type of intervention aiming at reducing acute hospital admission. Systematic reviews, randomised controlled trials, quasi randomised controlled trials, controlled before-after studies and interrupted time series were eligible study designs. The process of selecting studies, assessing them, extracting data and grading the total evidence was done by two researchers individually, with any disagreement solved by a third. We made use of meta-analyses from included systematic reviews, the remaining synthesis is descriptive. Based on the type of intervention, the included studies were categorised in: 1) Interventions to structure and standardise clinical practice, 2) Geriatric specialist services and 3) Influenza vaccination.

**Results:**

Five systematic reviews and five primary studies were included, evaluating a total of 11 different interventions. Fewer hospital admissions were found in four out of seven evaluations of structuring and standardising clinical practice; in both evaluations of geriatric specialist services, and in influenza vaccination of residents. The quality of the evidence for all comparisons was of low or very low quality, using the GRADE approach.

**Conclusions:**

Overall, eleven interventions to reduce hospital admissions from nursing homes were identified. None of them were tested more than once and the quality of the evidence was low for every comparison. Still, several interventions had effects on reducing hospital admissions and may represent important aspects of nursing home care to reduce hospital admissions.

## Background

Longevity, chronic illness, frailty and deficits in activities of daily living are common characteristics of the geriatric nursing home population. These features are predispositions to a trajectory of health with acute incidences which raises the question about acute care hospitalisation. Acute flares in nursing home residents’ health may call for services not necessarily available in the nursing homes, such as diagnostic procedures, particular interventions or a shift towards end-of-life care. Indeed, studies from a range of different countries with well-developed nursing home sectors have demonstrated that acute hospital admissions occur commonly, with annual rates from 9% up to 60% [1-7]. Noteworthy, large variations in hospital admission rates from nursing homes are not only observed between countries, but also within countries and in small geographic areas [1,8,9].

Adding to this picture, a number of studies have pointed to the detrimental impacts that hospitalisations may have on elderly people, including iatrogenic illnesses, like infections to functional and cognitive decline [10-16]. Additionally, the nursing home population is appointed to account for many potentially unnecessary hospitalisations, with estimates between 19–67% [17-20]. As such, a reduction of hospital admissions among nursing home residents may potentially serve a dual benefit of improving care for residents, as well as reducing use and monetary cost of specialist health care.

Although it is strongly communicated that nursing home residents represent an overuse of specialist services [17-20] it is not clear what strategies can best substitute hospitalisations. Thus, enforced by healthcare reforms that warrant for a shift in the provision of health care from specialist to primary care settings, there is an increasing interest for care models that can replace frequent and perhaps unnecessary use of hospital admissions from nursing homes [21,22]. Still, it is not clear what strategies can best substitute hospitalisations, to achieve the twofold aim of providing high quality services and reducing cost in specialist health care.

The objective of this systematic review is therefore to summarise the effects of interventions to reduce acute hospitalisations from nursing homes.

## Methods

This is an update of a systematic review published in Norwegian by the Norwegian Knowledge Centre for the Health Services [23]. A protocol for the first version, including eligibility criteria, search strategy and methods of analysis, was developed in advance and made available in PROSPERO [24].

### Eligibility criteria

We considered studies with a geriatric nursing home study population, evaluating any type of intervention aiming at reducing hospitalisation, compared to care as usual or a different intervention. The primary outcome measure of interest was acute hospital admission. The secondary outcomes, listed in the protocol, are only reported in the supplementary summary of findings tables (Additional file 1: Tables S4-S12). Study designs eligible for this review were systematic reviews, randomized controlled trials (RCT), quasi-randomized controlled trials, controlled before-after studies and interrupted time series. We imposed no restriction on language or publication year in the search. We decided to deal with languages as they emerged and to draw on language proficiency levels in the review group, among colleagues or to translate studies if necessary. The two studies in Spanish and Austrian was managed in the review team and no studies were excluded due to language.

### Literature search

The updated literature searches were carried out from the inception and until April 2013 in the following databases: The Cochrane Library, PubMed, MEDLINE Ovid 1946, EMBASE Ovid 1974, ISI Web of Science and CINAHL Ebsco. The search strategy was developed using keywords and standardised key words, where appropriate. The search terms derived from the population/setting (nursing home) and the primary outcome (hospitalisation). The complete search strategy is available in the (Additional file 1: Table S1) and in the protocol [24].

### Study selection and assessment

Titles and abstracts that the literature search brought fourth were screened independently by two researchers (LF, BG). Any potentially relevant publication was ordered in full-text and assessed for inclusion and exclusion according to eligibility criteria, following the same procedure. Any disagreement in the process of selecting, assessing and collecting data was solved by a third researcher (GJ).

Reviews that fulfilled criteria for inclusion were assessed for methodological quality using a check list based on international criteria for assessing reviews [25]. Only reviews of high quality were included. From the included SRs, we only used data from included primary studies that were relevant to our eligibility criteria. We used the review authors’ own assessment of risk of bias. For primary studies we used the risk of bias tool from Cochrane Handbook [26]. We used GRADE (Grading of Recommendations, Assessment, Development and Evaluation) to assess and grade the quality of the overall documentation for each outcome as high, middle, low or very low quality [27].

### Data extraction process

For each included study, we extracted the following information: Full reference, the number of study participants, type of intervention, type of control intervention, the setting and outcomes. If the outcome was measured several times in a study, we used the last observation.

### Synthesis of results

Where possible, we reported the overall effect estimate from meta-analyses in included systematic reviews (Additional file 1: Tables S11-S12) [28,29]. For the remaining included studies, analyses were descriptive, due to differences in interventions. We used RevMan 5 to recalculate estimates if we considered that this would improve the reporting of the effect estimates, the preferred presentation being relative risks (RR) with 95% confidence intervals (CI).

## Results

### Study selection

**Figure 1:**
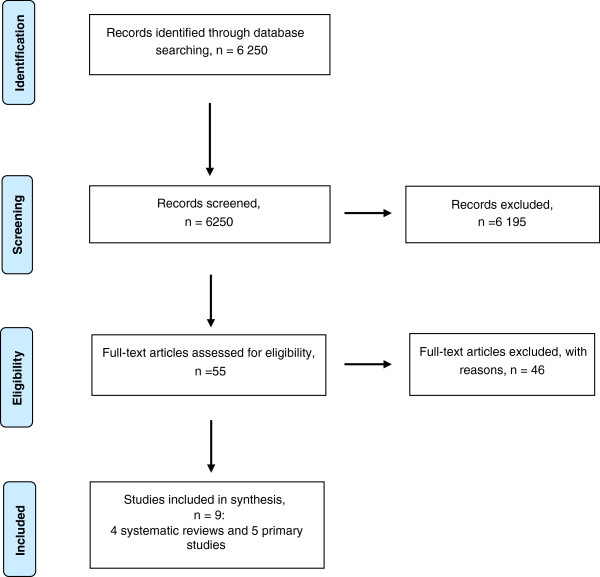
Flow chart of the selection process.

The literature search identified a total of 6 250 unique references. Of these, 54 studies were retrieved in full text and assessed according to eligibility criteria. A total of four systematic reviews and five primary studies met the inclusion criteria and were included. Figure 1 holds the details of the selection process. A table of excluded studies and reason for exclusion is available as an (Additional file 1: Table S2).

### Characteristics of included studies

Four systematic reviews and five primary studies, evaluating a total of 11 different interventions were included. All but two of the included studies were in English; these two were Austrian and Spanish [30,31]. Follow-up periods varied between 30 days up to 3 years. The interventions varied fundamentally and made it unfeasible to do meta analyses; the exception being two included Cochrane reviews on the effect of influenza vaccination [28,29].

We classified the type of interventions into three categories; *Interventions to structure or standardise clinical practice*, *geriatric specialist services* and *influenza vaccination*. The categories were decided after the inclusion of studies, to cluster studies according to type of intervention. The results are presented according to these categories: Tables 1, 2, 3 hold descriptions of included studies and Additional file 1: Tables S4-S12 are summary of findings.

**Table 1:** **Table of included studies in the category *Interventions to structure and standardise clinical practice***

Study, design, included studies if SR*	**Population**	**Intervention**	**Control**	**The setting and nationality**
**Robinson 2012**[32], Systematic review, 3/4 studies were relevant for inclusion	People with cognitive impairment	Defined as ‘Any kind of advance care planning’ by review authors.	Usual care	Any care environment, including nursing homes
Caplan 2006, Controlled before after design		A structured educational programme (Let Me Decide) for health personnel, residents and their families		Nursing homes in Canada, Australia and USA
Molloy 2000, Randomised controlled trial		The Let Me Decide programme in addition to hospital-to-the-nursing-home		
Morrison 2005, Non-randomised controlled trial		Half-day course for social workers in guiding residents and families in ACP. Feedback to physicians was given in intervention group to initiate referral to palliative care.		
**Hall 2011**[33], Systematic review	Residents of care homes for older people	Defined as ‘All types of palliative care service delivery interventions’ by review authors.	Not specified	Setting defined as ‘collective institutional settings where care is provided’ Nursing homes in USA
1/3 studies was relevant for inclusion: Casarett 2005, Randomised controlled trial				
**Hutt 2011**[34], Controlled before-after study	Nursing home residents with symptoms of systemic lower respiratory tract infection	Multifaceted implementation of a national guideline for management of nursing-home-acquired pneumonia	Usual care	16 nursing homes in Colorado, USA (8 intervention homes and 8 control homes)
**Loeb 2006**[35], Cluster randomised controlled trial	Nursing home residents with pneumonia	On-site treatment of pneumonia according to pathway	Usual care	22 nursing homes Ontario, Canada
**Lee 2002**[36], Cluster randomised controlled trial	Nursing home residents with chronic obstructive pulmonary disease	Community nurses followed up residents for 6 months post-hospitalisation according to a care protocol	Usual care	45 nursing homes in Hong Kong, China

*See included systematic review for full reference of included primary studies.

**Table 2:** **Table of included studies in the category *Geriatric specialist services***

**Study, design**	**Population**	**Intervention**	**Control**	**The setting and nationality**
**Díaz-Genúndez 2011**[30], Controlled before-after study	Nursing home residents	Ambulatory geriatric team doing comprehensive geriatric assessments of residents and revision of their medication, in addition to providing educational sessions and support to staff	Usual care	14 nursing homes in Spain (10 voluntary intervention homes, 14 control homes)
**Shippinger 2012**[31], Controlled before-after study	Nursing home residents	Mobile GEriatric Consultant geriatric service (GECO) in addition to usual care	Usual care	Two nursing homes in Austria (one intervention home and one control home)

**Table 3:** **Table of included studies in the category *Influenza vaccination***

Study, design, Included studies if SR*	**Population**	**Intervention**	**Control**	**The setting and nationality**
**Thomas 2010**[29], Systematic review	Healthcare workers caring for elderly residents in institutions	Promotion of vaccination of healthcare workers with any influenza vaccine given alone or with other vaccines	Usual care	Any type of institution for elderly, including nursing homes
2/5 studies were relevant for inclusion: Hayward 2006, Lemaitre 2009, Cluster-randomised controlled trials				Nursing homes in England and France
**Jefferson 2010**[28], Systematic review	Elderly people	Vaccination with any influenza vaccine	Usual care	Irrespective of setting, including nursing homes
One to 27 out of 75 studies were relevant: Feery 1976, Saah 1986b, Horman 1986, Fyson 1983a, Patriarca 1985a, Goodman 1982, Straburg 1986, Fyson 1983b, Meiklejohn 1987, Cartter 1990c, Cartter 1990a, Cartter 1990, Aylor 1992, Morens 1995, Monto 2001, Murayama 1999, Ruben 1974, Saah 1986a, Arroyo 1984, Coles 1992, Patriarca 1985b, Caminiti 1994, Deguchi 2001, Howells 1975a, Howells 1975b, Howells 1975c, Saah 1986c, Strassburg 1986, Arden 1988, Cartter 1990b, Taylor 1992, Mukerjee 1994, Isaacs 1997, Leung 2007, D’Alessio 1969, Currier 1988, Saito 2002a, Saito 2002b, Gross 1988, Cuneo Crovari 1980, Howarth 1987a, Howarth 1987b				Nursing homes in USA, Australia, Canada, Japan, Italy, China, UK

*See included systematic review for full reference of included primary studies.

Only the results for the primary outcome (hospitalisation) are reported in the manuscript. The results for other outcomes are included in the summary of finding tables (Additional file 1: Tables S4-S12).

### Methodological quality

Overall, using GRADE, we judged the quality of the evidence as being low or very low for all outcomes. All but one comparison was downgraded because of a high or unclear risk of bias. Imprecision was the second most frequent reason to downgrade and indirectness was a problem in several studies. The evidence from one of the systematic reviews was additionally downgraded due to inconsistency of results between studies. In the supplemental file, all judgements for assessing methodological quality are made explicit (Additional file 1: Tables S4-S12).

### Effects of interventions

#### Interventions to structure and standardise clinical practice

Seven different interventions in this category had been evaluated in two systematic reviews and three single studies (Table 1). One systematic review summarised the effect of advance care planning in people with cognitive impairment, and included three studies relevant for this review [32]. Two of the studies, a cluster randomised controlled trial and a controlled before after study, both investigated a structured program aimed at residents, families and health personnel in the intervention homes, but the latter additionally provided hospital-to-nursing-home services. Both studies found that intervention homes reported fewer hospitalisations than the control homes (mean 0.27 hospitalisations vs. 0.48, *p* = 0.001, and RR 0.89, 95% CI: 0.85-0.93, respectively). In the third study, a cluster-RCT, social workers in intervention wards received a course in how to do structured interviews with residents to identify needs for advance directives. The effect of this intervention on number of hospitalisations was unclear (RR 0.60, 95% CI 0.28-1.28) (Additional file 1: Table S4).

The other review evaluated the effectiveness of palliative care service delivery interventions in nursing homes, and one of three included studies met our eligibility criteria [33]. This was an RCT aimed at increasing the use of hospice services by supporting physicians in identifying residents in need for this. The intervention group reported lower hospitalisation rate (mean annual admissions 0.28 per bed (SD ± 0.70) vs. 0.49 (SD ± 0.89), *p* = 0.004) (Additional file 1: Table S5).

Hutt and colleagues [34] tested the effect of a multifaceted implementation strategy of a national guideline for management of nursing home acquired pneumonia in a cluster-RCT [34]. The risk difference between intervention and control group was a statistically non-significant reduction in hospitalisation for the intervention group (Additional file 1: Table S6). Loeb and colleagues [35] compared the use of a clinical care pathway to usual care for nursing home residents developing symptoms of lower respiratory infections, also using a cluster-RCT design (Additional file 1: Table S7) [35]. Among the intervention homes there was a statistically significant lower hospital admission rate (weighted mean difference of 12% (95% CI:5-18%, *p* = 0.001)). In the last of the three primary studies, Lee and colleagues [36] compared a care protocol with usual care for residents recently hospitalised with chronic obstructive pulmonary disease (COPD) (Additional file 1: Table S8) [36]. There was not a statistical significant difference in re-hospitalisation rates between the groups in number of COPD-related readmissions (*p*-value = 0.67).

The quality of the evidence for the results for this category was graded low or very low quality (Additional file 1: Tables S4-S8).

#### Geriatric specialist services

The use of geriatric specialist services in nursing homes was evaluated in two single studies [30,31]. Both of these tested the effectiveness of providing ambulant specialist services, in addition to usual care, but in different facets. Schippinger [31] evaluated a service where a physician did regular and on-call visits intended to provide services otherwise associated with hospitalisation (Additional file 1: Table S10) [31]. The intervention home had fewer cases of hospitalisation than the control home (6.1 cases vs. 11.7 cases per 100 residents, *p* < 0.01). Dìaz-Gegùndez [30] evaluated an ambulant team with a nurse and a physician, doing comprehensive geriatric assessments of residents as well as reviewing medications and providing support to staff (Additional file 1: Table S9) [30]. Also in this study, the intervention group reported fewer hospitalisations than the control group (56 cases vs 32 cases per 100) (RR 0.58, 95% CI: 0.52-0.65) (calculated by us, based on numbers given in the study).

The quality of the evidence for the results for this category was graded very low (Additional file 1: Tables S9-S10).

#### Influenza vaccination

Two Cochrane reviews concerning influenza vaccination were relevant for this review; one reviewing studies where *health personnel* were encouraged to vaccinate and another where effects of influenza vaccination among *residents* were reviewed [28,29]. In the first review by Thomas [29], two out of five included studies were relevant to us, but the effect of influenza vaccination in health personnel on hospitalisation of residents was unclear (RR 0.89, 95% CI: 0.75-1.06) (Additional file 1: Table S11) [29]. In the review by Jefferson [28], the meta-analysis showed a favourable effect on hospitalisation for the residents that were vaccinated (RR 0.51, 95% CI: 0.33-0.66) (1.1% in intervention group vs 1.7% in control group) (Additional file 1: Table S11) [28].

The quality of the evidence for the effect of vaccinating health personnel or nursing home residents was graded low and very low, respectively (Additional file 1: Tables S11-S12).

## Discussion

We set out to systematically review the effects of interventions to reduce acute hospital admissions from nursing homes. Four systematic reviews and five primary studies were included, evaluating a total of eleven different interventions. Overall, using GRADE, the quality of the evidence for all outcomes was low or very low. In systematic reviews, the quality of evidence reflects the extent of confidence that an estimate of effect is correct [37]. As such, our confidence in the findings is weak. Still, we believe that this review is an important contribution as the first truly systematic and transparent approach to the topic. Further, several of the included studies showed promising effects on hospital admission, but were downgraded, in many cases because of the relatively few included patients. Among the seven *interventions to structure or standardise treatment*, a reduction in hospital admissions was found for four of them. This was the case for two out of three advance care planning interventions, one intervention to enhance the use of palliative care services and one where a care pathway for lower respiratory tract infections was tested. For the three remaining interventions in this category; an ACP-intervention involving social workers, one multifaceted implementation of a national guideline for the treatment of pneumonia and a care protocol for residents with COPD, a statistical significant difference in hospitalisation between the intervention and control group was not found. Two single studies tested *geriatric specialist services,* both involving flexible and add-on special competence and human resources to the care in nursing homes. Both of these reported fewer hospitalisations in favour of the intervention. Two Cochrane reviews respectively tested *influenza vaccination* among residents and health personnel. The case of vaccinating residents, although many studies were identified, only observational design studies were found, making it infeasible to draw conclusive inferences from the findings. Also, noteworthy, all of the studies failed to show an effect on laboratory-confirmed influenza, raising serious doubt in the inherent conceptual mechanism of the intervention. Further, it is not clear whether promoting influenza vaccination among health personnel makes a difference on hospitalisations of nursing home residents.

### Limitations

Although the literature searches were conducted by a research librarian using well-developed search filters and strategies, there is always a possibility of missing relevant studies due to the structural complexity of the literature databases, lack of use of pregnant text words in abstracts and also, in some instances, inconsistent indexing of articles. In our search we required that the references should be either indexed with terms for hospitalisations or having used ‘hospitalisation’ or a synonym in the abstract.

The screening process introduced predicament for a few studies, where hospitalisation was an outcome measure but where the intervention was not aimed at reducing hospitalisations. In these cases hospitalisation was measured as a possible adverse effect of an intervention that, in turn, was not aimed at reducing hospitalisations. When in doubt, we used the aim of the study to determine whether the intervention could coherently impact on acute hospitalisation admissions. This may have led to different decisions in the hands of other reviewers.

Most often, the comparison of the intervention was against usual care, however, this can obviously have different meanings in various settings and usually the descriptions leave it somewhat unclear what the comparison really was. Caution must be shown when judging the transferability of findings and circumstances from one nursing home setting to another, particularly across nationalities.

### Implications and future research

The clinical usefulness of this review is weakened by the low quality of the evidence of the included studies, as well as the limited numbers of evaluations for each comparison. Unfortunately, this is not a stand-alone example in the sphere of research in nursing homes, as the body of evidence with robust designs to inform decisions is generally small, with few interventions evaluated more than once [38]. Several intervention studies were excluded because of a weak before-after study design, such as the INTERACT studies [39,40]. The fact that the quality of evidence for every comparison in this review was downgraded is not equivalent to claiming the interventions do not impact on hospitalisation, though. Rather, this renders the need for further studies, to increase the confidence of the findings.

As health care policies around the globe are seeking ways to increase efficacy and reduce strain on specialist services, reducing emergency admissions is often accentuated as the key to achieve this. However, it is currently debated whether the frail and old really represent much of a potential in this case [41-43]. Although remaining a target population in the health-policy discourse, it appears that much of the rhetoric is based on anecdotal arguments. This review brings together what is available evidence to inform the case for acutely ill nursing home residents. The fact that we found few studies fulfilling our eligibility criteria, even as accepting less rigorous designs for evaluating effectiveness of interventions, confirms that little research effort is placed on this matter. This is an evidence-policy gap with an urgent need to better inform current policies and reforms in the case of nursing home residents. A larger and better body of evidence is required before recommendations and incitements come in place. Moreover, research policies should request trials in the intersection between primary and secondary care for frail and old residents, emphasising which methodological demands are necessary for the research to have impact.

Most of the studies referred to introductorily, to underpin the argument for reducing hospitalisations, are based on observational studies [10-16], without control groups. Intuitively, reducing hospitalisations for this very frail group of elderly is favourable, but prospective studies with control groups are required to provide more solid evidence for the well-used arguments. Secondly, the studies where many hospitalisations are claimed to be ambulatory care sensitive, and thus potentially unnecessary, are mostly based on secondary analysis of administrative data [17-20]. These judgments are thus made in retrospect, where contextual information is lost.

For future studies evaluating interventions to reduce hospitalisations, adherence to the framework of complex interventions is recommended, where barriers and facilitators for treating the residents on-site, and process evaluations are addressed [44,45]. Clearly, the potential for interdisciplinary innovations across levels of health care is present, and necessary. It goes without saying, but interventions reducing hospitalisations must hold proof of being a more gentle option for the frail and old, in addition to being equally safe and effective.

## Conclusions

Few evaluations are conducted on the effects of interventions to reduce hospital admissions from nursing homes. Eleven evaluated interventions were identified, but none were tested more than once with a rigorous study design. Although the quality of evidence was low for all comparisons in this review, some of the interventions had effects on reducing hospital admissions. These interventions, such as advance care planning, palliative care, care pathways and geriatric specialist services, may represent important aspects of nursing home care to reduce hospital admissions and should be studied further. Our findings suggest an evidence-policy gap, where current policies and practices are lacking evidence-based management strategies to underpin them.

## Competing interests

All authors declare they have no competing interests.

## Authors’ contributions

BG, LF and GJ made substantial contributions to conception of the study and to the development of the protocol of this review, the acquisition of data and interpretation of the data. BG drafted the manuscript and LF and GJ has been involved in revising it critically before submission. Approval of the final version has been given from all authors.

## Pre-publication history

The pre-publication history for this paper can be accessed here:

http://www.biomedcentral.com/1472-6963/14/36/prepub

## Supplementary Material

Additional file 1 An additional file is available (Supplementary File), containing the complete search strategy (Table S1), table of excluded studies with reason for exclusion (Table S2), risk of bias assessments of primary studies (Table S3) and GRADE summary of findings tables (Table S4-S12). Click here for file
