# Supplementary material for: Reducing hospital admissions from nursing homes: a systematic review
Source: BMC Health Serv Res. 2014 Jan 24;14:36. doi: 10.1186/1472-6963-14-36 (PMC3906881; doi:10.1186/1472-6963-14-36)
Supplement: Additional file 1 — An additional file is available (Supplementary File), containing the complete search strategy (Table S1), table of excluded studies with reason for exclusion (Table S2), risk of bias assessments of primary studies (Table S3) and GRADE summary of findings tables (Table S4-S12). [file 1472-6963-14-36-S1.pdf]

CONTENTS – SUPPLEMENTAL FILE

		Page
Table S1:	Search strategy (Cochrane Library, Medline, Embase, Pubmed, Cinahl Ebsco, ISI Web of Science, ongoing studies)	2
Table S2:	Table of excluded studies with reason for exclusion	8
Table S3:	Risk of bias assessments of primary studies	12
GRADE summary of findings tables for interventions to structure and standardize care		18
Table S4:	GRADE Summary of findings table – Advance care directives	19
Table S5:	GRADE Summary of findings table – Palliative care	21
Table S6:	GRADE Summary of findings table – Multifaceted implementation of a national guideline for management of nursing home-acquired pneumonia	22
Table S7:	GRADE Summary of findings table – On-site treatment of pneumonia according to care pathway	23
Table S8:	GRADE Summary of findings table – Care protocol for hospitalized residents with COPD	24
GRADE summary of findings tables for geriatric specialist services interventions		25
Table S9:	GRADE Summary of findings table - Geriatric specialist team compared to usual care	25
Table S10:	GRADE Summary of findings table – Ambulant geriatric services compared to usual care	26
GRADE summary of findings tables for influenza vaccination		27
Table S11:	GRADED Summary of findings table – Influenza vaccination of nursing home residents	28
Table S12:	GRADE Summary of findings table – Influenza vaccination of health personnel	29

Table S1: Search strategy

– Cochrane Library – 15.6.2012 Updated search: April 2013

ID	Search	Hits
#1	MeSH descriptor Nursing Homes explode all trees	848
#2	MeSH descriptor Homes for the Aged, this term only	403
#3	(nursing NEXT (home* or facilit*)):ti,ab,kw	1741
#4	("home? for the aged" OR "home? for the elderly"):ti,ab,kw	19
#5	((intermediate or long-term or longterm) NEXT care facilit*):ti,ab,kw	267
#6	(#1 OR #2 OR #3 OR #4 OR #5)	1958
#7	MeSH descriptor Hospitalization explode all trees	10175
#8	(hospital NEAR/3 (treat* or stay or days or care)):ti,ab,kw	10344
#9	(stay NEAR/2 length):ti,ab,kw	7120
#10	(hospitaliz* or hospitalis* or rehospitalis* or rehospitaliz*):ti,ab,kw	14159
#11	((hospital? or patient?) NEAR/3 (admit* or admis* or readmit* or readmis* or transfer)):ti,ab,kw	3743
#12	MeSH descriptor Emergency Service, Hospital explode all trees	1418
#13	MeSH descriptor Emergency Medical Services, this term only	738
#14	((acute or immediate or emergency or critical) NEXT (care or service)):ti,ab,kw	3050
#15	"use of emergency department?":ti,ab,kw	8
#16	"emergency department? use":ti,ab,kw	25
#17	(trauma center*):ti,ab,kw or (trauma centr*):ti,ab,kw	780
#18	(#7 OR #8 OR #9 OR #10 OR #11 OR #12 OR #13 OR #14 OR #15 OR #16 OR #17)	31760
#19	(#6 AND #18)	399

– MEDLINE Ovid – 9.10.2012 Updated search: April 2013

Database(s): **Ovid MEDLINE(R) In-Process & Other Non-Indexed Citations, Ovid MEDLINE(R) Daily and Ovid MEDLINE(R)** 1946 to Present

#	Searches	Results
1	nursing homes/ or intermediate care facilities/ or skilled nursing facilities/	30369
2	Homes for the Aged/	10432
3	(nursing adj (home* or facilit*)).tw.	21831
4	(home? for the aged or home? for the elderly).tw.	2064
5	((intermediate or long-term or longterm) adj care facilit*).tw.	3602
6	or/1-5	43698
7	hospitalization/ or "length of stay"/ or patient admission/ or patient readmission/ or patient transfer/	132876
8	(hospital adj3 (treat* or stay or days or care)).tw.	86679

9	(stay adj2 length).tw.	23455
10	(hospitaliz* or hospitalis* or rehospitalis* or rehospitaliz*).tw.	135076
11	((hospital? or patient?) adj3 (admit* or admis* or readmit* or readmis* or transfer)).tw.	107590
12	emergency service, hospital/ or trauma centers/	43670
13	Emergency Medical Services/	29850
14	((acute or immediate or emergency or critical) adj (care or service)).tw.	32957
15	("use of emergency department?" or "emergency department? use" or trauma cent?r*).tw.	8557
16	or/7-15	439737
17	6 and 16	6913
18	(news or comment or editorial).pt.	882738
19	cochrane database of systematic reviews.jn.	9141
20	comment on.cm.	520922
21	or/18-20	891825
22	17 not 21	6741
23	(randomized controlled trial or controlled clinical trial).pt.	420053
24	(randomi?ed or randomly or trial or groups).tw.	1677302
25	or/23-24	1799994
26	Comparative Study/	1610942
27	("before and after" or comparison or compared or controlled or control group? or experimental or quasi* or (time adj (point? or series)) or repeated measur*).tw.	3566091
28	or/26-27	4462092
29	limit 22 to "reviews (best balance of sensitivity and specificity)"	573
30	25 or 28 or 29	5246651
31	22 and 30	2974

Embase Ovid – 8.10.2012 Updated search: April 2013

Database(s): Embase 1974 to 2012 June 13

#	Searches	Results
1	nursing home/	38607
2	nursing home patient/	2007
3	home for the aged/	10429
4	(nursing adj (home* or facilit*)).tw.	26626
5	(home? for the aged or home? for the elderly).tw.	2685
6	((intermediate or long-term or longterm) adj care facilit*).tw.	4381
7	or/1-6	54123
8	hospitalization/	180165
9	"length of stay"/	67273
10	hospital admission/	93230
11	hospital readmission/	11014
12	patient transport/	17535

13	(hospital adj3 (treat* or stay or days or care)).tw.	117295
14	(stay adj2 length).tw.	33619
15	(hospitaliz* or hospitalis* or rehospitalis* or rehospitaliz*).tw.	186955
16	((hospital? or patient?) adj3 (admit* or admis* or readmit* or readmis* or transfer)).tw.	150174
17	emergency health service/	60467
18	((acute or immediate or emergency or critical) adj (care or service)).tw.	45061
19	("use of emergency department?" or "emergency department? use" or trauma cent?r*).tw.	10060
20	or/8-19	650330
21	7 and 20	9138
22	(systematic review or literature review).ti.	44522
23	"cochrane database of systematic reviews".jn.	3773
24	or/22-23	48290
25	21 not 24	9082
26	Randomized Controlled Trial/	332844
27	Controlled Clinical Trial/	392529
28	Multicenter Study/	102047
29	(randomis* or randomiz* or randomly or random allocat*).tw. or groups.ab.	1898563
30	(trial or multicentre or multicenter or multi centre or multi center).ti.	167265
31	or/26-30	2168574
32	limit 25 to "reviews (best balance of sensitivity and specificity)"	872
33	comparative study/	685675
34	("before and after" or comparison or compared or controlled or control group? or experimental or quasi* or (time adj (point? or series)) or repeated measur*).tw.	4338163
35	or/33-34	4722461
36	31 or 32 or 35	5746049
37	25 and 36	3628
38	limit 37 to embase	2658

PubMed – 20.6.2012 Updated search: April 2013

Search	Query	Items found
#34	Search (#32) AND #33	32
#33	Search publisher[sb]	409897
#32	Search (#30) AND #31	4437
#31	Search randomised OR randomized OR "random allocation" OR "randomly allocated" OR trial OR multicenter OR "multi-center" OR multicentre OR "multi-centre" OR "systematic review"	1103708
#30	Search (#9) AND #29	27322
#29	Search ((((((((#10) OR #15) OR #16) OR #17) OR #19) OR #22) OR #23) OR #24) OR #28	1456446
#28	Search "emergency departments" OR "emergency department"	42290
#24	Search "acute care" OR "immediate care" OR "emergency care" OR "critical care" OR "acute service" OR "immediate service" OR "emergency service" OR "critical service"	116593
#23	Search "trauma center" OR "trauma centers" OR "trauma centre" OR "trauma centres"	12137
#22	Search "emergency medical services" OR "emergency medical service"	32109
#19	Search "hospital emergency service" OR "hospital emergency services"	37047
#17	Search (hospital OR hospitals OR patient OR patients) AND (admit* or admis* or readmit* or readmis* or transfer)	260164
#16	Search hospitaliz* OR hospitalis* OR rehospitalis* OR rehospitaliz*	175146
#15	Search "stay length" OR "length of stay" OR "stay lengths" OR "lengths of stay"	61854
#10	Search (hospital AND (treat* OR stay OR days OR care))	1121755
#9	Search ((((#2) OR #3) OR #6) OR #7) OR #8	92930
#8	Search "long-term care facility" OR "long-term care facilities"	3340

#7	Search "longterm care facility" OR "longterm care facilities"	13
#6	Search "intermediate care facility" OR "intermediate care facilities"	712
#3	Search "home for the aged" OR "homes for the aged" OR "home for the elderly" OR "homes for the elderly"	75905
#2	Search "nursing home" OR "nursing homes" OR "nursing facility" OR "nursing facilities"	37371

Cinahl Ebsco – 1.10.2012 Updated search: April 2013

#	Query	Results
S42	S23 and S41	599
S41	S24 or S25 or S26 or S27 or S28 or S29 or S30 or S31 or S32 or S33 or S34 or S35 or S36 or S37 or S38 or S39 or S40	952243
S40	AB systematic review	6266
S39	TI systematic review	8650
S38	PT systematic review	25772
S37	MH systematic review	11487
S36	TI ((intervention* or controlled or "control group*" or compare or compared or comparison or before N5 after or pre N5 post or pretest or "pre test" or posttest or "post test" or quasi* or experimental or evaluat* or effect or impact or "time series" or "time point*" or “repeated measur*”)) OR AB ((intervention* or controlled or "control group*" or compare or compared or comparison or before N5 after or pre N5 post or pretest or "pre test" or posttest or "post test" or quasi* or experimental or evaluat* or effect or impact or "time series" or "time point*" or "repeated measur*"))	552294
S35	TI (randomis* or randomiz* or random* W0 allocat*) OR AB (randomis* or randomiz* or random* W0 allocat*)	65813
S34	(MH "Health Services Research")	5917
S33	(MH "Multicenter Studies")	6126
S32	(MH "Quasi-Experimental Studies+")	6255
S31	(MH "Pretest-Posttest Design+")	19439
S30	(MH "Experimental Studies")	11821
S29	(MH "Nonrandomized Trials")	127
S28	(MH "Intervention Trials")	4367
S27	(MH "Clinical Trials")	75603
S26	(MH "Randomized Controlled Trials")	11399
S25	PT research	754684
S24	PT clinical trial	51541
S23	S7 and S22	4051
S22	S8 or S9 or S10 or S11 or S12 or S13 or S14 or S15 or S16 or S17 or S18 or S19 or S20 or S21	149937
S21	trauma cent#r*	4152
S20	"emergency department? use"	7
S19	"use of emergency department*"	119
S18	((acute or immediate or emergency or critical) N1 (care or service))	73717
S17	MH Emergency Medical Services	13656
S16	MH trauma centers	2285
S15	MH emergency service	21466
S14	((hospital* or patient*) N3 (admit* or admis* or readmit* or readmis* or transfer))	23804
S13	(hospitaliz* or hospitalis* or rehospitalis* or rehospitaliz*)	34321
S12	(stay N2 length)	17351
S11	(hospital N3 (treat* or stay or days or care))	18866
S10	MH patient admission	6385

S9	MH "length of stay"	13659
S8	MH hospitalization	10519
S7	S1 or S2 or S3 or S4 or S5 or S6	35815
S6	((intermediate or long-term or longterm) N1 "care facilit*")	2343
S5	(home* for the aged or home* for the elderly)	1451
S4	(nursing N1 (home* or facilit*))	34151
S3	MH Nursing Home Patients	7801
S2	MH skilled nursing facilities	1772
S1	MH nursing homes	13657

- ISI Web of Science – 8.10.2012 Updated search: April 2013
-

Set	Results	
# 21	1,410	#20 AND #16
# 20	2,882,383	#19 OR #18 OR #17
# 19	1,961,058	Topic=("comparative stud*") OR Topic=(("before and after" or “control group?” or experimental or quasi* or (time NEAR (point? or series)) or “repeated measur*”))
# 18	38,378	Topic=("systematic review*")
# 17	993,848	Topic=(randomised or randomized or "random allocation" or "randomly allocated" or trial or multicenter or "multi center" or multicentre or "multi centre")
# 16	6,014	#15 AND #4
# 15	274,061	#14 OR #13 OR #12 OR #11 OR #10 OR #9 OR #8 OR #7 OR #6 OR #5
# 14	375	Topic=("emergency department$ use")
# 13	44	Topic=("use of emergency department$")
# 12	73,667	Topic=(((acute or immediate or emergency or critical) NEAR (care or service)))
# 11	6,959	Topic=((("trauma center*" or "trauma centr*")))
# 10	3,247	Topic=("Emergency Medical Services")
# 9	88	Topic=(("hospital emergency service"))
# 8	45,724	Topic=((((hospital? or patient?) NEAR/3 (admit* or admis* or readmit* or readmis* or transfer))))
# 7	103,662	Topic=(((hospitaliz* or hospitalis* or rehospitalis* or rehospitaliz*)))
# 6	28,342	Topic=(((stay NEAR/2 length)))
# 5	84,360	Topic=(((hospital NEAR/3 (treat* or stay or days or care))))
# 4	31,170	#3 OR #2 OR #1
# 3	2,767	Topic=((((intermediate* or long-term or longterm) NEAR "care facilit*")))
# 2	148	Topic=(("home$ for the aged" or "home$ for the elderly"))
# 1	29,494	Topic=((nursing NEAR (home* or facilit*)))

Search for ongoing studies:

Search date: 11 April 2013

Prospero: http://www.crd.york.ac.uk/prospero/

nursing homes

17 hits, 1 possibly relevant

http://apps.who.int/trialsearch/default.aspx

*nursing homes AND hospital**

37 hits, none relevant

http://clinicaltrials.gov

nursing homes

403 hits, 5 possibly relevant

Table S2: Excluded studies with reason for exclusion

Study references	Cause for exclusion of study
Abrahm JL. Advances in palliative medicine and end-of-life care. Annu Rev Med 2011;62:187-99.	Not an experimental study.
Ackermann RJ, Kemle KA. The effect of a physician assistant on the hospitalization of nursing home residents. JAGS 1998;46:610-614.	No control group.
Adema HH. Oseltamivir in the treatment and prevention of influenza in the nursing home. Tijdschrift voor Verpleeghuisgeneeskunde 2004;28(3):15-8.	Primary study excluded, systematic review included (Jefferson 2010).
Ahearn DJ, Jackson TB, Mcllmoyle J, Weatherburn AJ. Improving end of life care for nursing home residents: an analysis of hospital mortality and readmission rates. Postgrad Med J 2010;86:131-135.	Not an experimental study.
Beghe C. An advance directive programme in nursing homes reduced services use without affecting patient satisfaction. EBN 2000;3:125.	A review of Molloy 2000.
Bellantonio S, Kenny AM, Fortinsky RH, Kleppinger A, Robison J, Gruman C, et al. Efficacy of a geriatrics team intervention for residents in dementia-specific assisted living facilities: effect on unanticipated transitions. J Am Geriatr Soc 2008;56(3):523-8.	Population does not satisfy inclusion criterion.
Bollig G, Husebø BS, Husebø S. Vakttjeneste for leger på sykehjem. Tidsskr Nor Legeforen 2008;128:2722-2724.	No control group.
Booy R, Lindley RI, Dwyer DE, Yin JK, Heron LG et al. Treating and preventing influenza in aged care facilities: a cluster randomised controlled trial.PLOS ONE 2012;7(10):1-16	Not an aim to reduce hospitalisation.
Bowman CE, Elford J, Dovey J, Campbell S, Barrowclough H. Acute hospital admissions from nursing homes: some may be avoidable. Postgrad Med J 2001;77:40-42.	Retrospective study design.
CARE program replaces pre-admission assessment... Client Assessment, Referral, and Evaluation. Kans Nurse 1994;69(10):11.	Not a scientific article.
Crilly J, Chaboyer W, Wallis M, Thalib L, Polit D. An outcomes evaluation of an Australian Hospital in the Nursing Home admission avoidance programme. J Clin Nurs 2010;20(7-8):1178-87.	Retrospective study design.
Deguchi Y, Takasugi Y, Tatara K. Efficacy of influenza vaccine in the elderly in welfare nursing homes: reduction in risks of mortality and morbidity during an influenza A (H3N2) epidemic. J Med Microbiol 2000;49(6):553-6.	Excluded primary study and included systematic review in stead (Jefferson 2010).

Deguchi Y, Takasugi Y. Efficacy of influenza vaccine in the elderly: reduction in risks of mortality and morbidity during an influenza A (H3N2) epidemic for the elderly in nursing homes. Int J Clin Lab Res 2000;30(1):1-4.	Excluded primary study and included systematic review in stead.
Dempsey CL. Nursing home-acquired pneumonia: outcomes from a clinical process improvement program (Structured abstract). Pharmacotherapy 1995;15:S33-S38.	Not an experimental study.
Hancook HC, Close H, Mason JM, Murphy JJ, Fuat A, de Belder M et al. Feasibility of evidence-based diagnosis and management of heart failure in older people in care: a pilot randomised controlled trial. BMC Geriatrics 2012, 12:70.	Intervention aim not to reduce hospitalisation. Feasibility study.
Hayward AC, Harling R, Wetten S, Johnson AM, Munro S, Smedley J, et al. Effectiveness of an influenza vaccine programme for care home staff to prevent death, morbidity, and health service use among residents: cluster randomised controlled trial. BMJ 2006;333(7581):1241.	Excluded primary study and included systematic review in stead.(Thomas 2010).
Hutt E, Ruscin JM, Corbett K, Radcliff TA, Kramer AM, Williams EM, Liebrecht D, Klenke W, Hartmann S. A mulitifaceted intervention to implement guidelines improved treatment of nursing home-acquired pneumonia in a State Veterans Home. JAGS 2006;54:1694-1700.	Pilot test.
Kane RA, Lum TY, Cutler LJ, Degenholtz HB, Yu T-C. Resident outcomes in small-house nursing homes: A longitudinal evaluation of the Initial Green House Program. J Am Geriatr Soc 2007;55(6):832-9.	Not a program for reducing hospitalisation.
Kane RL, Keckhafer G, Flood S, Bershadsky B, Siadaty MS. The effect of Evercare on hospital use. J Am Geriatr Soc 2003;51(10):1427-34.	Retrospective study design.
Kane RL, Flood S, Bershadsky B, Keckhafer G. Effect of an innovative medicare managed care program on the quality of care for nursing home residents. Gerontologist 2004;44(1):95-103.	Retrospective study design.
Kane RL, Garrard J, Skay CL. Radpsevocj D;. Buchanan JL, McDermott SM, Arnold SB, Kepperle L. Effects of a Geriatric Nurse Practitioner on Process and Outcome of Nursing Home Care. American Journal of Public Health 1989;79:1271-1277.	Retrospective study design.
Kane RL, Garrard J, Buchanan JL, Rosenfeld A, Skay C, McDermott S. Improving primary care in nursing homes. JAGS 1991;39:359-367.	Retrospective study design.
Konetzka RT, Spector W, Limcangco MR. Reducing hospitalizations from long-term care settings. Med Care Res Rev 2008;65(1):40-66.	Not a systematic review.
Krüger K, Jansen K, Grimsmo A, Eide GE, Geitung JT. Hospital admissions from nursing homes: rates and reasons. Nursing research and practice 2011. Article ID 247623, 6 pages, doi:10.1155/2011/247623	Not an experimental study

Loeb M, Brazil K, Lohfeld L, McGeer A, Simor A, Stevenson K, et al. Effect of a multifaceted intervention on number of antimicrobial prescriptions for suspected urinary tract infections in residents of nursing homes: cluster randomised controlled trial. BMJ 2005;331(7518):669.	Intervention aim not to reduce hospitalisation.
Mennnick F. Treating pneumonia in nursing home residents. American Journal of Nursing 2006;106:22.	Review of Loeb 2006.
Miller SC, Gozalo P, Mor V. Hospice enrollment and hospitalization of dying nursing home patients. Am J Med 2001;111:38-44.	Retrospecitve study design.
Mor V, Intrator O, Fries BE, Phillips C, Teno J, Hiris J, et al. Changes in hospitalization associated with introducing the Resident Assessment Instrument. J Am Geriatr Soc 1997;45(8):1002-10.	No control group.
Mott PD, Barker WH. Hospital and medical care use by nursing home patients: the effect of patient care plans. J Am Geriatr Soc 1988;36(1):47-53.	No control group.
Naughton BJ, Mylotte JM, Ramadan F, Karuza J, Priore RL. Antibiotic use, hospital admissions, and mortality before and after implementing guidelines for nursing home-acquired pneumonia. JAGS 2001;49:1020-1024.	Implementation of guidelines for antibiotic treatment of nursing-home acquired pneumonia.
Oliver DP, Porock D, Zweig S. End-of-life care in U.S. nursing homes: A review of the evidence. J AM MED DIR ASSOC 2005;6(3 SUPPL.):S20-S30.	Review of studies in general about end-of-life care. No relevant studies.
Ouslander JG, Lamb G, Tappen R, Herndon L, Diaz S, Roos BA, et al. Interventions to reduce hospitalizations from nursing homes: evaluation of the INTERACT II collaborative quality improvement project. J Am Geriatr Soc 2011;59(4):745-53.	The comparison group was not selected beforehand.
Pilgram EH, Hierzer A, Bohnstingl M, Osprian I, Schippinger W. The GECO project: impact of a geriatric consultant service on acute hospital admissions of nursing home residents. 8th Congress of the EUGMS/European Geriatric Medicine 2012; 35:S110 (P259).	Conference abstract.
Rainfray M, Bourdel-Marchasson I, Dehail P, Richard-Harston S. Comprehensive geriatric assessment: A useful tool for prevention of acute situations in the elderly. Ann Med Interne (Paris) 2002;153(6):397-402.	Only about the assessment tool.
Snider KT, Snider EJ, Johnson JC, Hagan C, Schoenwald C. Preventative osteopathic manipulative treatment and the elderly nursing home resident: a pilot study. J Am Osteopath	Not an aim to reduce hospitalisation.

Assoc 2012;112(8):489-501.	
Spinewine A, Fialová D, Byrne S. The role of the pharmacist in optimizing pharmacotherapy in older people. Drugs Aging 2012;29(6):495-510.	Not a systematic review.
Stevenson DG, Bramson JS. Hospice care in the nursing home setting: a review of the literature. J Pain Symptom Manage 2009;38(3):440-51.	Not a systematic review.
Teno J, Lynn J, Connors AF, Wenger N, Phillips RS, Alzola C, et al. The illusion of end-of-life resource savings with advance directives. J Am Geriatr Soc 1997;45(4):513-8.	Re-analysis of data collected from other studies.
Teno J, Lynn J, Wenger N, Phillips RS, Murphy DP, Connors AF, et al. Advance directives for seriously ill hospitalized patients: Effectiveness with the Patient Self-Determination Act and the SUPPORT intervention. J Am Geriatr Soc 1997;45(4):500-7.	Population does not satisfy inclusion criterion.
Teno JM, Gozalo P, Mitchell S, Bynum J, Dosa D, Mor V. Does increasing advance care planning reduce terminal hospitalizations among nursing home residents? Palliative Medicine 2010;24:S5-S229.	Conference abstract.
Teno JM, Gozalo P, Michell S, Bynum J, Dosa D, Mor V. Terminal hospitalizations of nursing home residents: does facility increasing the rate of do not resuscitate orders reduce them?	Retrospective study design.
Werner RM, Konetzka RT, Stuart EA, Norton EC, Polsky D, Park J. Impact of public reporting on quality of postacute care. HSR: Health Services Research 2009;44:1169-1187.	Retrospective study design.
Yayan J. Trends in intensive care in patients over 90 years of age. Clinical Interventions in Aging 2012;7:339-347.	Retrospective study design.
Ye Y, Phippis A, Reiman S, Carr D, Parker R. Implementing INTERACT (Intervention to Reduce Avoidable Acute Care Transfer) Distance Learning Curriculum to Reduce Avoidable Acute Care Transfer and Improve the Quality of Care in a Skilled Nursing Facility. J AM MED DIR ASSOC 2012;13(3):B15.	Conference abstract.
Zafirau WJ, snyder SS, Hazelett SE, Bansal A, McMahon S. Improving transitions: efficacy of a transfer form to communicate patients' wishes. American Journal of Medical Quality 2012;27:291-296.	Not to reduce hospitalisations but to improve transitions.

Table S3. Risk of bias assessments of primary studies

Hutt 2011<br>**Domain**	**Support for judgement**	**Review authors' judgement**
Selection bias		
**Random sequence generation**	No random sequence generation. "… there were significant differences between intervention and control homes in baseline guideline compliance, subject characteristics and facility characteristics, including nursing resident staffing ratios and nursing staff turnover."	No
**Allocation concealment**	"Denver-area homes received the intervention; homes in Kansas and Missouri served as controls"	No
Performance bias		
**Blinding of participants and personnel** *Assessments should be made for each main outcome (or class of outcomes).*	"unblinded study", but the experiment and control groups were in different geographic areas. We assess the risk of bias to be low.	Yes
Detection bias		
**Blinding of outcome assessment** *Assessments should be made for each main outcome (or class of outcomes).*	Data collected from chart review by 6 trained nurses using a "previously tested, systematic chart review instrument on laptop computers […] with built-in range and logic checks". Every tenth chart was re-reviewed by the project manager and another data collector.	Yes
Attrition bias		
**Incomplete outcome data** *Assessments should be made for each main outcome (or class of outcomes).*	The authors state that "the appropriateness of nearly one third of the hospitalization decisions could not be evaluated because a full set of vital signs was not recorded at illness onset". Also, they report that "Only items with inter-rater reliability scores of 0.7 or better by Cohen's Kappa or percent agreement are reported here".	Unclear
Reporting bias		
**Selective reporting**	No reason to suspect.	Yes
**Other sources of bias**		Unclear
**Overall judgment**	High risk of bias	

**Dìaz-Gegùndez 2011**<br>Domain	Support for judgement	Review authors' judgement
Selection bias		
**Random sequence generation**	No random sequence generation due to study design, CBA.	NO
**Allocation concealment**	Probably no allocation concealment due to study design, CBA. It is not reported how/why the group of 10 nursing homes was chosen for the intervention and the other 14 as control.	NO
Performance bias		
**Blinding of participants and personnel** *Assessments should be made for each main outcome (or class of outcomes).*	Not possible to blind participants and personnel. Is it likely that intervention homes withhold residents from hospital because there is a strong focus on this due to the study going on? Recruiters most likely not blinded.	Unclear
Detection bias		
**Blinding of outcome assessment** *Assessments should be made for each main outcome (or class of outcomes).*	Hospitalisation is an objective measure – not likely that unblinded outcome assessors make a difference.	Yes
Attrition bias		
**Incomplete outcome data** *Assessments should be made for each main outcome (or class of outcomes).*	How was outcome assessment done? No loss is reported.	Unclear
Reporting bias		
**Selective reporting**	No reason to suspect.	
**Other sources of bias**	A small difference between baseline values for outcome measure. Group characteristics at baseline are not reported. Control of confounding not reported.	No
**Overall judgment**	High risk of bias	

**Loeb 2006**<br><br>Domain	Support for judgement	Review authors' judgement
Selection bias		
**Random sequence generation**	"Nursing homes were paired by the number of occupied beds to help ensure similar rates of pneumonia and other lower respiratory tract infections [...]. One member of each pair was randomized to [...] using a random numbers table."	Yes
**Allocation concealment**	"...by a statistician independent of the study team ..." (pp 2504).	Yes
Performance bias		
**Blinding of participants and personnel** *Assessments should be made for each main outcome (or class of outcomes).*	It was not possible to blind participants or personnel. The clinical pathway that was to be implemented was standardised in a rather clear and unambiguous float chart with very specific assessments of criteria for hospitalisation or not. We assess risk of bias as low.	Yes
Detection bias		
**Blinding of outcome assessment** *Assessments should be made for each main outcome (or class of outcomes).*	We perceive the outcomes to be assessed, hospitalization and death, as objective outcomes.	Yes
Attrition bias		
**Incomplete outcome data** *Assessments should be made for each main outcome (or class of outcomes).*	The authors declare 97% follow-up of hospitalization (p 2506)	Yes
Reporting bias		
**Selective reporting**	No reason to suspect	Yes
**Other sources of bias**	This was a matched cluster trial. Clustering was accounted for, but somewhat unclear whether the matching was accounted for.	Unclear
**Overall judgment**	Low risk of bias	

**Lee 2002**<br><br>Domain	Support for judgement	Review authors' judgement
Selection bias		
**Random sequence generation**	How the sequence was generated is not reported	Unclear
**Allocation concealment**	Cluster trial	Yes
Performance bias		
**Blinding of participants and personnel** *Assessments should be made for each main outcome (or class of outcomes).*	We assess risk of bias here to be low, in spite of participants and community nurses most likely were not blinded.	Yes
Detection bias		
**Blinding of outcome assessment** *Assessments should be made for each main outcome (or class of outcomes).*	Likely not blinded, but objective data: hospital days	Yes
Attrition bias		
**Incomplete outcome data** *Assessments should be made for each main outcome (or class of outcomes).*	They lost ca 20% after randomization due to death, returning home or moving to another nursing home. Not possible to see how this is distributed between groups.	Unclear
Reporting bias		
**Selective reporting**	No reason to suspect	Yes
**Other sources of bias**	Cluster trial: Blinding of recruiters not reported.<br>Statistical analyses does not correct for cluster effect or for matching.	No
**Overall judgment**	High risk of bias	

**Schippinger 2012**<br><br>Domain	Support for judgement	Review authors' judgement
Selection bias		
**Random sequence generation**	No random sequence generation due to study design CBA – one intervention home and one control home	No
**Allocation concealment**	Probably no concealment of allocation due to study design CBA – as above. We do not know how/why one nursing homes was chosen for the intervention and the other as control.	No
Performance bias		
**Blinding of participants and personnel** *Assessments should be made for each main outcome (or class of outcomes).*	Not possible to blind any of the two. This could influence the decision to hospitalize. Recruiters most likely not blinded.	Unclear
Detection bias		
**Blinding of outcome assessment** *Assessments should be made for each main outcome (or class of outcomes).*	Most likely not blinded, but objective data: acute hospitalisations.	Yes
Attrition bias		
**Incomplete outcome data** *Assessments should be made for each main outcome (or class of outcomes).*	No loss is reported.	Unclear
Reporting bias		
**Selective reporting**	No reason to suspect.	Yes
**Other sources of bias**	No big differences between baseline values for outcome measure or baseline characteristics	Yes
**Overall judgment**	**High risk of bias**	

GRADE summary of findings tables for interventions to structure and standardise care

Table S4 GRADE summary of findings table – Advance care directives

TABLE S4: EDUCATIONAL INTERVENTION ALONE OR IN COMBINATION WITH OTHER INTERVENTIONS TO ENHANCE ADVANCE CARE PLANNING

Population: Nursing home residents
Setting: Nursing homes, Canada, Australia, USA
Intervention: Educational intervention alone or in combination with other interventions
Comparison: Usual care

Educating staff, residents and family in advance care planning compared to usual care

Outcome	Comparing risk in the two groups (95%CI)		Relative effect (95% CI)	Number of participants (studies)	Quality of documentation (GRADE)	Comment
	Assumed risk	Compared risk				
	**Usual care**	**Educational intervention alone in Advance Care Planning**				
**Hospitalisation per resident** Follow-up 18 months	Mean number of hospital admissions per resident in control group was 0,48	Mean number of hospital admissions per resident in intervention group was 0,21 lower (CI not reported)		6 clusters/1133 participants (1)	⊕⊕⊖⊖ **Low**[1,2]	'Intervention nursing homes reported fewer hospitalisations per resident (mean 0.27 vs 0.48 = -0.21, *p*=0.001))'
**Mortality** Follow-up 18 months	**276 per 1000**	**237 per 1000**	RR 0,86 (0.70–1.04)	6 clusters/1133 participants (1)	⊕⊕⊖⊖ **Low**[1,2]	Not-significant finding (24% vs 28%, *p*=0.20)

Educating staff, residents and health personnel in advance care planning combined with hospital-in-the-nursing-home compared to usual care

Outcome	Comparing risk in the two groups (95%CI)		Relative effect (95% CI)	Number of participants (studies)	Quality of documentation (GRADE)	Comment
	Assumed risk	Compared risk				
	**Usual care**	**Educating health personnel in Advance Care Planning combined with hospital-in-the-nursing- home.**				
**Hospital admission** Follow-up: 3 years	Not reported	Not reported	RR 0.89 (0.85-0.93)	32 nursing homes/514 participants (1)	⊕⊖⊖⊖ **Very low**[2,5]	Controlled before-after study
**Mortality** Follow-up 3 years	See comment	See comment		32 nursing homes/514 participants (1)	⊕⊖⊖⊖ **Very low** [2,5]	30.4 versus 41.6 deaths per 100 nursing home beds (*p*=0.0425)

Educating social workers in advance care planning combined with feedback to physician, compared to usual care

Outcome	Comparing risk in the two groups (95%CI)		Relative effect (95% CI)	Number of participants (studies)	Quality of documentation (GRADE)	Comment
	Assumed risk	Compared risk				

	Usual care	Educating social workers in ACP combined with feedback to physician			
**Hospital admission**	**271 per 1000**	**163 per 1000**<br>(76 to 347)	**RR 0.60**<br>(0.28 -1.28)	4 clusters/139 participants<br>(1)	⊕⊖⊖⊖<br>**Very low**[2,3,4]

Table S5: GRADE Summary of findings table – Palliative care

TABLE S5: INTERVENTION TO IMPROVE PALLIATIVE CARE IN NURSING HOMES

Population: Nursing home residents
Setting: Nursing homes, USA
Intervention: Increase the use of hospice services in nursing homes
Comparison: Usual care

**Outcome**	**Comparing risk in the two groups (95 % CI)** Assumed risk	Compared risk	**Relative effect (95% CI)**	**Number of participants (studies)**	**Quality of documentation (GRADE)**	**Comment**
	**Usual care**	**Use of palliative care services**				
**Hospitalisation** Follow-up 6 months	Mean hospitalisation rate in the control group was **0.49**	Mean hospitalisation rate in the intervention group was **0.21 lower (CI not reported)**		205 (1)	⊕⊖⊖⊖ **Very low**[1,2]	'Intervention residents also had fewer acute admissions (mean [SD]: 0.28 [0.70] vs 0.49 [0.89]; *p*=0.04 [Wilcoxon rank sum test])'
**Length of stay in hospital**	Mean length of stay in the control group was **3.0 days**	Mean length of stay in the intervention group was **1.8 days lower (CI not reported)**		205 (1)	⊕⊖⊖⊖ **Very low**[1,2]	'Intervention residents also […] spent fewer days in an acute care setting (mean [SD]: 1.17 [3.32] vs 2.99 [6.07]; *p*=0.03 [Wilcoxon rank sum test])'
**Mortality** Follow-up: 6 months	82 per 1000	140 per 1000 (62 to 316)	RR 1.72 (0.76 to 3.87)	205 (1)	⊕⊖⊖⊖ **Very low**[1,2]	Absolute risk reduction -5.86 % (CI -14.38 - 2.67).

[1] No concealment of allocation, no blinding, incomplete reporting of data, possible contamination between intervention and control groups as the doctors treated residents in both groups.
[2] Only one study with few events.

Table S6: GRADE Summary of findings table – Multifaceted implementation of national guideline for management of nursing home-acquired pneumonia

TABELL S6: MULTIFACETED IMPLEMENTATION OF A NATIONAL GUIDELINE FOR MANAGEMENT OF NURSING HOME-ACQUIRED PNEUMONIA COMPARED TO USUAL CARE

Patient: Nursing home residents
Setting: Nursing homes, USA
Intervention: Educational intervention with support of change agents for implementing a national guideline in the care of nursing-home acquired pneumonia
Comparison: Usual care

Outcome	Comparison of risk in the two groups (95 % CI)		Relative effect (95% CI)	Number of participants (studies)	Quality of documentation (GRADE)	Comment
	Assumed risk<br>**Usual care**	Compared risk<br>**Multifaceted intervention of a national guideline for management of nursing home-acquired pneumonia**				
**Hospitalization rate**<br><br>Follow-up: 3 years	Mean hospitalisation rate in the control group was 23 **%**	Mean hospitalisation rate in intervention group was 13,6 % (see comment)		16 nursing homes/ 1123 participants (1)	⊕⊖⊖⊖<br>**Very low**[1,2]	Controlled before-after study<br><br>Risk difference by the end of study-period, adjusted for differences at baseline = -2.9 (2.9 % fewer residents hospitalized in the intervention group, but this is a non-significant finding).

[1] No randomisation procedure, resulting in differences at baseline between intervention and control groups. No concealment of allocation. Unclear outcome reporting.
[2] Not possible to read how the authors have calculated the hospitalisation rates. It is unclear whether there is loss to follow-up for this outcome.

Table S7: GRADE Summary of findings table – On-site treatment of pneumonia according to care pathway

TABELL S7: Effect of a standardised care pathway for on-site treatment of pneumonia compared to usual care.

Population: Nursing home residents
Setting: Nursing homes, Canada
Intervention: Standardised care pathway for nursing home residents with lower respiratory infection
Comparison: Usual care

Outcome	Comparing risk in the two groups (95 % CI)		Relative effect (95% CI)	Number of participants (studies)	Quality of documentation (GRADE)	Comment
	Assumed risk<br>**Usual care**	Compared risk<br>**Care pathway for residents with lower respiratory infection**				
**Hospitalisation, proportion of groups**<br>Follow-up: Up to 30 days	Proportion hospitalised in control groups was **20 %**	Proportion hospitalised in intervention groups was **12 % lower** (5 to 18 lower)		22 nursing homes/ 661participants (1)	⊕⊕⊖⊖ **Low**[1]	'Adjusting for the clustering of residents in nursing homes, the weighted mean admission rate was 8% in the clinical pathway group vs 20% in the usual care group, with a weighted mean difference of 12% (95% confidence interval [CI], 5%-18%; =0.001)'.
**Length of stay in hospital (number of days)**<br>Follow-up: Up to 30 days	Mean length of stay in hospital in control groups was 1.74 days	Mean length of stay in hospital in intervention groups was **0.95 days lower** (0.34 to 1.55 lower)		22 nursing homes/ 661participants (1)	⊕⊕⊖⊖ **Low**[1]	'The mean number of hospital days per resident was 0.79 in the clinical pathway group vs 1.74 in the usual care group, with a weighted mean difference of 0.95 days per resident (95% CI, 0.34-1.55 days; *p*=0.004)'
**Mortality, percentage**<br>Follow-up: Up to 30 days.	Mean mortality in control group was 6 %	Mean mortality in intervention group was **2.9 % lower** (7.9 lower to 2.0 higher)		22 nursing homes/ 661participants (1)	⊕⊕⊖⊖ **Low**[1]	Death, %, 3.1 (−0.2 to 6.4) in intervention group vs 6.0 (1.8 to 10.3) in control group (difference of 2.9% (95% CI: -2.0-7.9, *p*-value = 0.23)).
**Time to normal vital signs**<br>Follow-up: Up to 30 days	Mean number of days to normal vital signs in control group was **2.66 days**	Mean number of days to normal vital signs in intervention group was **0.12 lower** (1.02 lower to 0.78 higher)		22 nursing homes/ 661participants (1)	⊕⊕⊖⊖ **Low**[1]	'2.55 (95% CI:1.60 to 3.48) days in pathway group vs 2.66 (95% CI: 2.24 to 3.08) in control group (difference of 0.12 days (95% CI: −0.78 to 1.02, *p*-value = 0.79)'

[1] Only one study with few events.

Table S8: GRADE Summary of findings table – Care protocol for hospitalized residents with COPD

TABLE S8: CARE PROTOCOL COMPARED TO USUAL CARE FOR NURSING HOME RESIDENTS WITH COPD

Population: Nursing home residents with COPD
Setting: Nursing homes, Hong Kong
Intervention: Care protocol
Comparison: Usual care

Outcome	Comparison of risk in the two groups (95% CI)		Relative effect (95% CI)	Number of participants (studies)	Quality of documentation (GRADE)	Comment
	Assumed risk<br>**Usual care**	Compared risk<br>**Care protocol**				
**Re-hospitalisations related to COPD**<br>Follow-up: 6 months	Mean number of re-hospitalisations in the control group was<br>**1.39**	Mean number of re-hospitalisations in the intervention group was<br>**0.15 higher**<br>(0.53 lower to 0.83 higher)		45 nursing homes/89 participants (1)	⊕⊖⊖⊖<br>**Very low**[1,2,3]	Total number of COPD-related readmissions: Mean 1.54 (SD± 1.75) in intervention group, 1.39 (SD± 1.51) in control group.
**Length of stay in hospital**<br>Follow-up: 6 months	Mean length of stay in control group was<br>**14.98 days**	Mean length of stay for intervention group was<br>**0.63 lower**<br>(8.87 lower to 7.61 higher)		45 nursing homes/89 participants (1)	⊕⊖⊖⊖<br>**Very low** [1,2,3]	Total number of COPD-related hospital days: Mean 14.35 (SD± 19.27) in intervention group; 14.98 (SD± 20.18) in control group.

[1] Method of sequence generation is not reported. 20 % attrition post-randomisation is not fully accounted for. Blinding of health personnel recruiting participants is not reported. Statistical analyses not adjusted for cluster-effect or matching.
[2] Uncertain about directedness for population and intervention.
[3] Only one study and very few events.

GRADE summary of findings tables for geriatric specialist services

Table S9: GRADE Summary of findings table - Geriatric specialist team compared to usual care

TABLE S9: Geriatric specialist team compared to usual care

Population: Nursing home residents
Setting: Nursing homes, Spain
Intervention: Geriatric specialist team
Comparison: Usual care

Outcome	Comparison of risk in the two groups (95% CI)		Relative effect (95% CI)	Number of participants (studies)	Quality of documentation (GRADE)	Comment
	Assumed risk<br>**Usual care**	Compared risk<br>**Geriatric specialist team**				
**Hospitalisation**<br>Follow-up: 3 years	**558 per 1000**	**324 per 1000**<br>(290 to 363)	**RR 0,58**<br>(0.52 to 0.65)	2057 beds<br>(1)	⊕⊖⊖⊖<br>**Very low** [1]	Controlled before-after design

[1] No sequence generation or concealed allocation. Attrition not accounted for. No baseline characteristics of the two groups. No adjustment for prognostic or possibly confounding factors.

Table S10: GRADE Summary of findings table – Ambulant geriatric services compared to usual care

TABLE S10: AMBULANT GERIATRIC SERVICE COMPARED TO USUAL CARE IN NURSING HOMES

Patient: Nursing home residents
Setting: Nursing homes, Austria

Intervention: Ambulant geriatric service
Comparison: Usual care

Outcome	Comparing risk in the two groups (95 % CI)		Relative effect (95 % CI)	Number of participants (studies)	Quality of documentation (GRADE)	Comment
	Assumed risk<br>**Usual care**	Compared risk<br>**Mobile geriatric team**				
**Hospitalisation**<br><br>Follow-up: 10 months.	Mean number of hospital admissions in the control group was **11.7 hospital admissions per 100 residents per month**	Mean number of hospital admissions in the intervention group was **5.6 lower** (CI not reported)		261 (1)	⊕⊖⊖⊖ **Very low**[1]	Controlled before-after study. In the intervention homes, the frequency of hospital admissions was 6,1 admissions per month per 100 residents, in the control group the number was 11,7 (*p*-value < 0.01)

[1] No random sequence generation or allocation of concealment. Only one intervention home and one control home. It is not reported how these homes were selected. No loss to follow-up is reported. The two nursing homes differ greatly in size.

GRADE summary of findings tables for Influenza Vaccination

Table S11: GRADED Summary of findings table – Influenza vaccination of nursing home residents

TABLE S11: INFLUENZA VACCINATION FOR NURSING HOME RESIDENTS COMPARED TO USUAL CARE

Population: Nursing home residents

Setting: Nursing homes, USA, Australia, Canada, Japan, Italy, China, Great Britain
Intervention: Influenza vaccination of nursing home residents
Comparison: Usual care

Outcome	Comparison of risk in the two groups (95 % CI)		Relative effect (95 % CI)	Number of participants (studies)	Quality of documentation (GRADE)	Comment
	Assumed risk	Compared risk				
	**Usual care**	**Influenza vaccination of nursing home residents**				
**Influenza like illness**<br>Follow-up: From 1 week to an influenza season	**264 per 1000**	**200 per 1000**<br>(174 to 232)	**RR 0.76**<br>(0.66 to 0.88)	12388<br>(26 studies)	⊕⊖⊖⊖<br>**Very low**[1,2,3]	
**Influenza, laboratory-confirmed**<br>Follow-up: from 6 weeks to 7 months	**63 per 1000**	**41 per 1000**<br>(20 to 82)	**RR 0.65**<br>(0.32 to 1.29)	1941<br>(8 studies)	⊕⊖⊖⊖<br>**Very low** [1,3]	
**Pneumonia**<br>Follow-up: from 3 weeks to 6 months	**7 per 1000**	**4 per 1000**<br>(3 to 5)	**RR 0.53**<br>(0.43 to 0.66)	10274<br>(17 studies)	⊕⊖⊖⊖<br>**Very low**[1]	
**Hospitalisation for influenza-like illness or pneumonia** Follow-up: from 1 week to 5 months	**17 per 1000**	**9 per 1000**<br>(5 to 14)	**RR 0.51**<br>(0.32 to 0.81)	28032<br>(12 studies)	⊕⊖⊖⊖<br>**Very low**[1]	
**Death caused by influenza or pneumonia**<br>Follow-up: 1-6 months	**11 per 1000**	**5 per 1000**<br>(4 to 7)	**RR 0.46**<br>(0.33 to 0.63)	32179<br>(27 studies)	⊕⊖⊖⊖<br>**Very low**[1]	
**Overall mortality**<br>Follow-up: 6 months	**177 per 1000**	**71 per 1000**<br>**(37** to **136)**	**RR 0.40** (0.21 to 0.77)	305<br>(1 study)	⊕⊖⊖⊖<br>**Very low**[1,4]	

[1] Possible selection bias
[2] Inconsistency of results across studies, heterogeneity I^2=57-60 %

[3] Wide confidence intervals
[4] Only one study with few events

Table S12: GRADE Summary of findings table – Influenza vaccination of health personnel

TABLE S12: PROMOTING INFLUENZA VACCINATION AMONG HEALTH PERSONNEL IN NURSING HOMES COMPARED TO USUAL CARE

Population: Nursing home residents
Setting: Nursing homes in France and England
Intervention: Influenza vaccination of health personnel
Comparison: Usual care

Outcome	Comparison of risk in the two groups (95% CI)		Relative effect (95% CI)	Number of participants (studies)	Quality of documentation (GRADE)	Comment
	Assumed risk	Compared risk				
	**Usual care**	**Influenza vaccinating health personnel**				
**Influenza-like illness** Follow-up: 118 and 145 days	**122 per 1000**	**88 per 1000** (76 to 102)	**RR 0.72** (0.62 to 0.84)	5972 (2)	⊕⊕⊖⊖ **Low**[1,2]	
**Hospitalization** Follow-up: 118 and 145 days	**96 per 1000**	**85 per 1000** (72 to 101)	**RR 0.89** (0.75 to 1.06)	5972 (2)	⊕⊕⊖⊖ **Low**[1,2]	
**Death caused by pneumonia** Follow-up: 118 days	**7 per 1000**	**11 per 1000** (5 to 23)	**RR 1.54** (0.75 to 3.17)	3400 (1)	⊕⊖⊖⊖ **Very low**[1,2]	
**Death caused by influenza-like illness** Follow-up: 145 days	**14 per 1000**	**10 per 1000** (5 to 21)	**RR 0.72** (0.35 to 1.47)	2572 (1)	⊕⊖⊖⊖ **Very low**[1,2]	
**Overall mortality**	**101 per 1000**	**74 per 1000** (61 to 90)	**RR 0.73** (0.60 to 0.89)	5972 (2)	⊕⊕⊖⊖ **Low**[1,2]	
**Influenza, laboratory-confirmed**						This outcome is not reported

[1] Unclear risk of bias (one of the studies not blinded, the other unclear. One study non-satisfactory treatment of missing data).
[2] Wide confidence intervals.
